# A novel framework for classification of two-class motor imagery EEG signals using logistic regression classification algorithm

**DOI:** 10.1371/journal.pone.0276133

**Published:** 2023-09-08

**Authors:** Rabia Avais Khan, Nasir Rashid, Muhammad Shahzaib, Umar Farooq Malik, Arshia Arif, Javaid Iqbal, Mubasher Saleem, Umar Shahbaz Khan, Mohsin Tiwana

**Affiliations:** 1 Department of Mechatronics Engineering, National University of Sciences & Technology, Islamabad, Pakistan; 2 Robot Design and Development Lab, National Centre of Robotics and Automation (NCRA), Punjab, Pakistan; Effat University, SAUDI ARABIA

## Abstract

Robotics and artificial intelligence have played a significant role in developing assistive technologies for people with motor disabilities. Brain-Computer Interface (BCI) is a communication system that allows humans to communicate with their environment by detecting and quantifying control signals produced from different modalities and translating them into voluntary commands for actuating an external device. For that purpose, classification the brain signals with a very high accuracy and minimization of the errors is of profound importance to the researchers. So in this study, a novel framework has been proposed to classify the binary-class electroencephalogram (EEG) data. The proposed framework is tested on BCI Competition IV dataset 1 and BCI Competition III dataset 4a. Artifact removal from EEG data is done through preprocessing, followed by feature extraction for recognizing discriminative information in the recorded brain signals. Signal preprocessing involves the application of independent component analysis (ICA) on raw EEG data, accompanied by the employment of common spatial pattern (CSP) and log-variance for extracting useful features. Six different classification algorithms, namely support vector machine, linear discriminant analysis, k-nearest neighbor, naïve Bayes, decision trees, and logistic regression, have been compared to classify the EEG data accurately. The proposed framework achieved the best classification accuracies with logistic regression classifier for both datasets. Average classification accuracy of 90.42% has been attained on BCI Competition IV dataset 1 for seven different subjects, while for BCI Competition III dataset 4a, an average accuracy of 95.42% has been attained on five subjects. This indicates that the model can be used in real time BCI systems and provide extra-ordinary results for 2-class Motor Imagery (MI) signals classification applications and with some modifications this framework can also be made compatible for multi-class classification in the future.

## Introduction

Brain-Computer Interface is a technology that creates a communication channel between the human brain and the external devices by picking up brain signals and translating them into artificial outputs. This system includes collecting data from the human brain, processing it to detect the user’s intent, and then training the system to actuate an external device.

Electroencephalography is a non-invasive technique that records brain signals by recognizing the change in brain wave patterns. The EEG signal is often an amalgamation of many base frequencies known to describe the cognitive, affective, or attentional states. These frequencies are based on particular ranges or bands. The EEG signal frequency range is 0–100 Hz, which is divided into five bands delta ‘δ’ (0.5–4 Hz), theta ‘θ’ (4–7 Hz), alpha ‘α’ (8–13 Hz), beta ‘β’ (13–30 Hz), and gamma ‘γ’ (within and above 35 Hz) [1]. The μ frequency band overlaps with α frequency band, but the first arises in the sensorimotor cortex while the second originates in the occipital and posterior regions of the brain [2].

Brain signals can be recorded non-invasively by various techniques such as functional magnetic resonance imaging (fMRI), magnetoencephalography (MEG) and electroencephalography (EEG), etc., as well as invasively through electrocorticography (ECoG) and microelectrode arrays (MEAs) [3]. For motor imagery (MI) data, EEG is mostly preferred due to its non-invasiveness, low cost, portability, less sensitivity to movement, and good temporal resolution [4].

The brain activity due to MI shows amplitude changes in certain frequency bands, also referred to as variations in sensorimotor rhythms. When a voluntary movement is performed, there is a decrease in amplitude, referred to as event-related desynchronizations (ERD), and after the activity is over, there is an increase in amplitude known as event-related synchronizations (ERS) [5]. The ERD and ERS are known as event-related potential (ERP). The MI-related EEG signals originating in the sensorimotor region of the brain are based on μ (8–12 Hz) and β (14–30 Hz) frequency bands [6, 7].

Brain signals are recorded from different brain regions, but directly using the EEG signals from all the channels would increase noise interference and may decrease the classification performance. Common spatial pattern (CSP) [8] is used to separate the appropriate signal characteristics from raw EEG data and represent them in a form interpretable by a human or a computer. Independent component analysis (ICA) [9] is a common approach for artifact removal. For identifying human brain activity patterns and translating them into commands, classification of EEG data is required. Various classification techniques such as support vector machine (SVM) [10], k-nearest neighbor (k-NN) [11], linear discriminant analysis (LDA) [12], naïve Bayes [13], decision trees [14], and logistic regression [15] are widely used.

The research is mainly motivated by the idea to present a balanced and optimized framework that classifies MI signals with a higher accuracy without compromising the execution time, which is a key factor for the successful implementation of any framework on real time BCI devices. This research involves the pre-processing techniques that removes noise and unwanted signals from the data effectively, feature extraction methods that extracts the optimum number of features without making the system complex and thus contributes to the literature by providing a combination of pre-processing and feature extraction techniques that improves the classification with reduced complexity.

Aiming to improve the accuracy of EEG signals classification, a new framework has been put forward in this research, to improve the classification accuracy of binary class EEG data, using a channel selection technique, employing a combination of Butterworth bandpass filter and ICA for pre-processing, and CSP & log-variance for feature extraction, along with different classification techniques such as SVM, LDA, naïve Bayes, decision trees, k-NN, and logistic regression. This allowed us to choose the most relevant classifier to obtain a pronounced improvement in the average classification accuracy of both datasets as compared to the approaches proposed earlier.

The remaining paper is structured as follows: Section II describes the literature review for all the previous work done on the chosen datasets, Section III describes the EEG data paradigm for both the datasets, section IV describes the proposed method; pre-processing, feature extraction, and classification, section V describes the results, section VI discusses the findings and the key factors that contributed in obtaining those results, section VII highlights the conclusion, followed by section VIII that shows the acknowledgement.

## Literature review

The proposed framework is tested on BCI Competition IV dataset 1 and BCI Competition III dataset 4a. Previously, researchers have used various techniques to classify BCI competition IV dataset 1 and BCI Competition III dataset 4a with commendable accuracies. Miao, Yangyang, *et al*. used regularized CSP (RCSP) for feature extraction and AdaBoost classifier for classification and attained an average classification accuracy of 78.4% on BCI competition IV dataset 1 [16]. On the same dataset, Qian, L., *et al*. used CSP for computing spatial filters, Stockwell transform to get time-frequency information, and CNN for classification and attained an average accuracy of 81.22% when the rectified linear activation function (ReLU) was used and an accuracy of 81.34% when the exponential linear unit activation function (ELU) was employed [17], Park, Y., *et al*. employed local region frequency optimized CSP (LRFCSP) and SVM and got an average classification accuracy of 84.7% [18], Fu, Rongrong, *et al*. used RCSP combined with RDA and achieved a maximum average accuracy of 87.21% [19] while recently, Zhang, *et al*. used CSP-Wavelet+LOG with FLDA as a classifier and achieved an average accuracy of 88.86% [20].

On BCI Competition III dataset 4a, Arabshahi, R., *et al*. employed CNN and Stacked Auto Encoders and attained an average classification accuracy of 82.0% [21]. Miao, Y., *et al*. used CTFSP to extract sparse CSP features and SVM with RBF to identify the MI task and attained an average accuracy of 85.0% on the same dataset [22], while Chen, S., *et al*. used CSP & SVM and pulled off an average classification accuracy of 86.86% [23] and Kirar, S. K., *et al*. attained an average classification accuracy of 90.58% by using graph theory and Quantum Genetic Algorithm followed by the employment of CSP and SVM [24]. Wijaya, A., *et al*. suggested a feature extraction technique based on logistic regression and two-stage detection (TSD) in the channel instantiation approach and attained an accuracy of 95.21% on this dataset [25]. There is still room for improvement in the accuracies of both datasets. For optimizing the classification accuracies, we have proposed a new methodology.

As seen above, the previous research work done can be summarized into two main categories: first group includes the methods that require initial raw data to be really precise and with minimal noise. The second group of methods involves complex frameworks with most of them having two or three stage data pre-processing and classification which consequently increases the computational complexity which is not feasible for real time applications.

So there is a need of a simple yet effective framework that can provide good results without increasing the complexity of the model.

## EEG data description

Two public MI datasets have been used in this study to evaluate the efficacy of the proposed framework.

### Dataset 1

**Fig 1:**
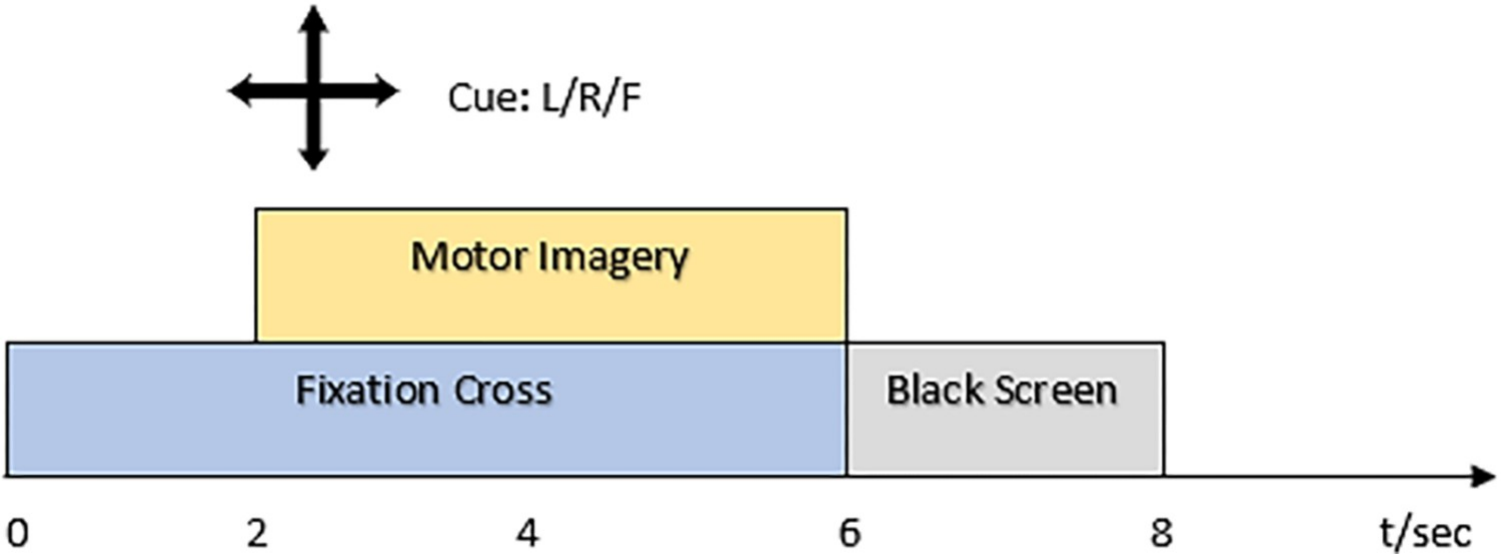
Timeline of a single trial for dataset 1.

The first dataset used in this work is dataset 1 from BCI competition IV provided by the Berlin BCI group [26]. This data has been collected from 59 channels and is recorded from seven healthy subjects namely, a, b, c, d, e, f, g. The sampling frequency of this dataset is 100 Hz. There is a total of three classes of motor imagery tasks involved; left hand, right hand, and foot, out of which any two classes have been performed for each subject. Visual cues in the form of arrows have been shown on the screen for 4 seconds and the subject has performed a certain mental task based on the cue shown, interleaved with 2s of fixation cross and 2s of a blank screen The fixation cross was displayed for 6 seconds while being superimposed on the cues. So the length of each trial is 4s followed by a rest period of 4s. Every subject has a total of 200 trials, 100 trials for each of the two selected classes for that certain subject. The length of a trial is 4s and the number of samples in each trial is 400 (4s x 100Hz). The data paradigm is shown in Fig 1.

### Dataset 2

**Fig 2:**
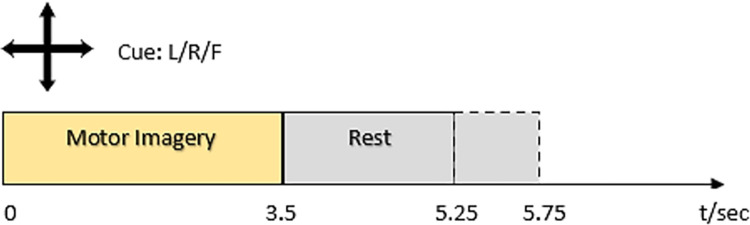
Timeline of a single trial for dataset 2.

The second dataset used in this work is dataset 4a of BCI Competition III. It comprises binary class MI EEG data collected from 5 healthy subjects namely, aa, av, al, ay, and aw, using 118 EEG channels positioned according to a 10–20 electrode system. Data of 280 trials have been collected for each subject. During each trial, every subject has been shown a 3.5s visual cue depicting three motor imagery tasks including left hand, right hand, and right foot. However, only the cues for the classes ’right hand’ and ’right foot’ have been provided for the competition. The signals have been bandpass filtered between 0.05 and 200 Hz, digitized at 1000 Hz, and then down-sampled to 100 Hz. The data paradigm is shown in Fig 2.

## Materials and methods

**Fig 3:**
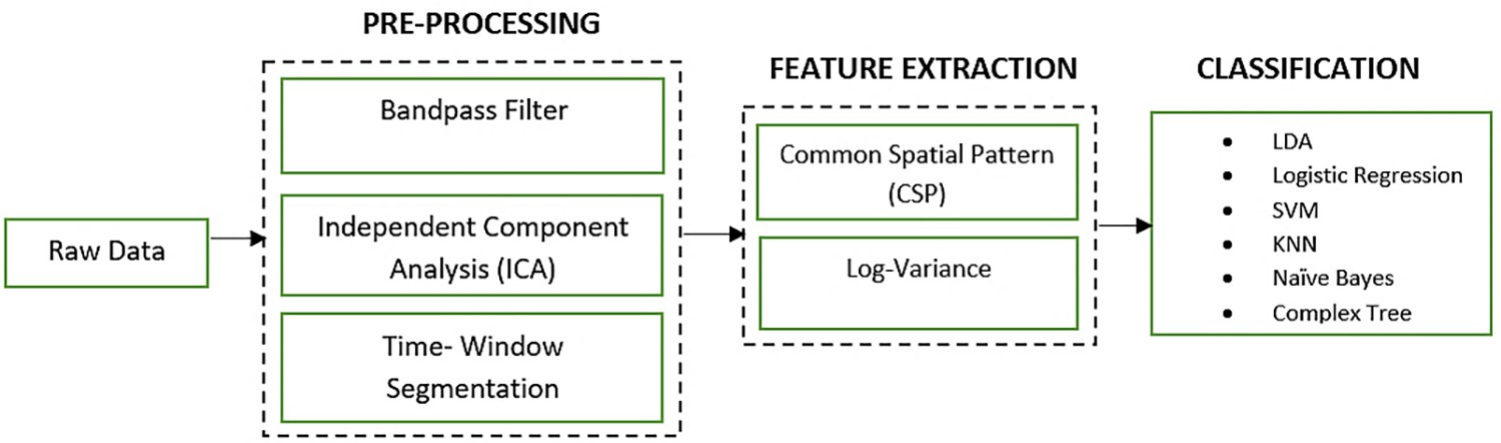
Block diagram of proposed methodology.

Fig 3 illustrates the flow of the proposed method. It includes data preprocessing, feature extraction, and classification, which will be discussed in detail in the preceding sections.

### Data preprocessing

Data preprocessing involves the filtering of raw EEG data into a suitable format for further analysis. The EEG signals acquired from the human scalp are not generally an accurate reflection of the actual signals originating from the brain as they contain a great deal of noise and artifacts. So for the separation of required signals, preprocessing techniques are employed.

### Filtering

After removing the outermost channels of the headset, a Butterworth bandpass filter is used to decompose the EEG signals into an 8–15 Hz frequency band to remove interference from EOG and EMG sources [27]. This frequency band exhibits the maximum indication of motor imagery.

### Independent Component Analysis (ICA)

For removing biological artifacts from data, ICA is widely used [28]. ICA enables effective source estimation, and thus plays a very important role in extracting useful information from raw EEG data [29]. ICA is a statistical technique that assumes non-Gaussian signal distribution [30] to separate a mixture of unknown signals into statistically independent components depending on the characteristics of the data.

The ICA generative model [31] is given by (1).


Y=AS (1)

Where, *Y* is the data matrix [*a*×*b*], where *a* denotes the number of samples, whereas *b* represents the number of variables to be measured respectively, and *A* is the matrix indicating the linear combination of source activities, S, to construct the input data matrix *Y*.

The ICA source activities, S, also referred to as independent components can be evaluated by taking the product of the input data matrix *Y* with the inverse matrix *W* of the matrix *A* [31] as shown in (2).


S=WY (2)

If the source signals are independent and have non-Gaussian distributions, ICA generates more accurate results [32].

### Time window segmentation

The time window chosen to extract the data of each trial for both dataset 1 and dataset 2 is 0.5 s to 2.5 s. The selected time window has been used by previous researchers [33, 34] on the same datasets as they provided better classification accuracy as compared to the other time windows.

## Feature extraction

### Common Spatial Pattern (CSP)

CSP is a powerful feature extraction technique commonly used for binary classification problems [35]. CSP can effectively extract the features of ERS and ERD in the motor imagery signals, so it has been widely used in BCI systems [36]. CSP maximizes the variance of spatially filtered signals for one class and minimizes it for the other to distinguish the features of both the classes, thus separating the classes by their variances.

The averaged normalized covariance matrices ${\bar{C}}_{a}$ and ${\bar{C}}_{b}$ are calculated for the binary class dataset by first calculating the normalized spatial covariance matrices *C*_*a*_ and *C*_*b*_, and then averaging them over all the trials for each class. A composite covariance matrix, *C*, is then obtained by the addition of the two normalized spatial covariance matrices [37] as given in (3).


$$C={\bar{C}}_{a}+{\bar{C}}_{b}=U_{0}\Sigma U_{0}^{T}$$ (3)

Where, *U*_*o*_ represents the eigenvectors and *Σ* denotes the diagonal matrix of the corresponding eigenvalues.

A whitening transformation matrix, *P*, is obtained by summing the eigenvalues and the eigenvectors of this composite covariance matrix, *C* [37] using (4).


$$P=\Sigma^{-\frac{1}{2}}U^{T}$$ (4)

P matrix transforms the normalized spatial covariance matrices ${\bar{C}}_{a}$ and ${\bar{C}}_{b}$ into another space [37], as shown in (5) and (6). The sum of the corresponding eigenvalues is always equal to 1 [37], as given by (7).


$$S_{a}=P{{\bar{C}}_{a}P}^{T}$$ (5)

$$S_{b}=P{{\bar{C}}_{b}P}^{T}$$ (6)

∑a+∑b=1 (7)

The spatial covariance matrices form a common eigenvector, *Q*, which then along with the whitening transformation matrix, P, forms a spatially filtered signal, W, [37] as given in (8).


$$W=U^{T}P$$ (8)

If the data has *n* number of channels then the matrix W has *[n x n]* dimensions, where the rows and the columns represent the number of channels and their corresponding spatial patterns, respectively. These spatial patterns help in the recognition of maximum and minimum values of the weighted channel that truncates surplus and repetitive data and reduces the dimensions of the data by neglecting the remaining values.

The use of two or three CSP filters from both ends of the eigenvectors is commonly recommended [36]. In this study, m = 3 has been chosen, i.e. six spatial filters have been used which means the first and the last three columns of the projection matrix have been selected as they give the maximum variance between both classes. The spatially filtered signal is then multiplied with the original data of each trial of both the classes to obtain the desired signal in the new subspace [37], as shown in (9) and (10).


$$Z_{a}=W_{2m}^{T}X_{a}$$ (9)

$$Z_{b}=W_{2m}^{T}X_{b}$$ (10)

### Log—variance

**Fig 4:**
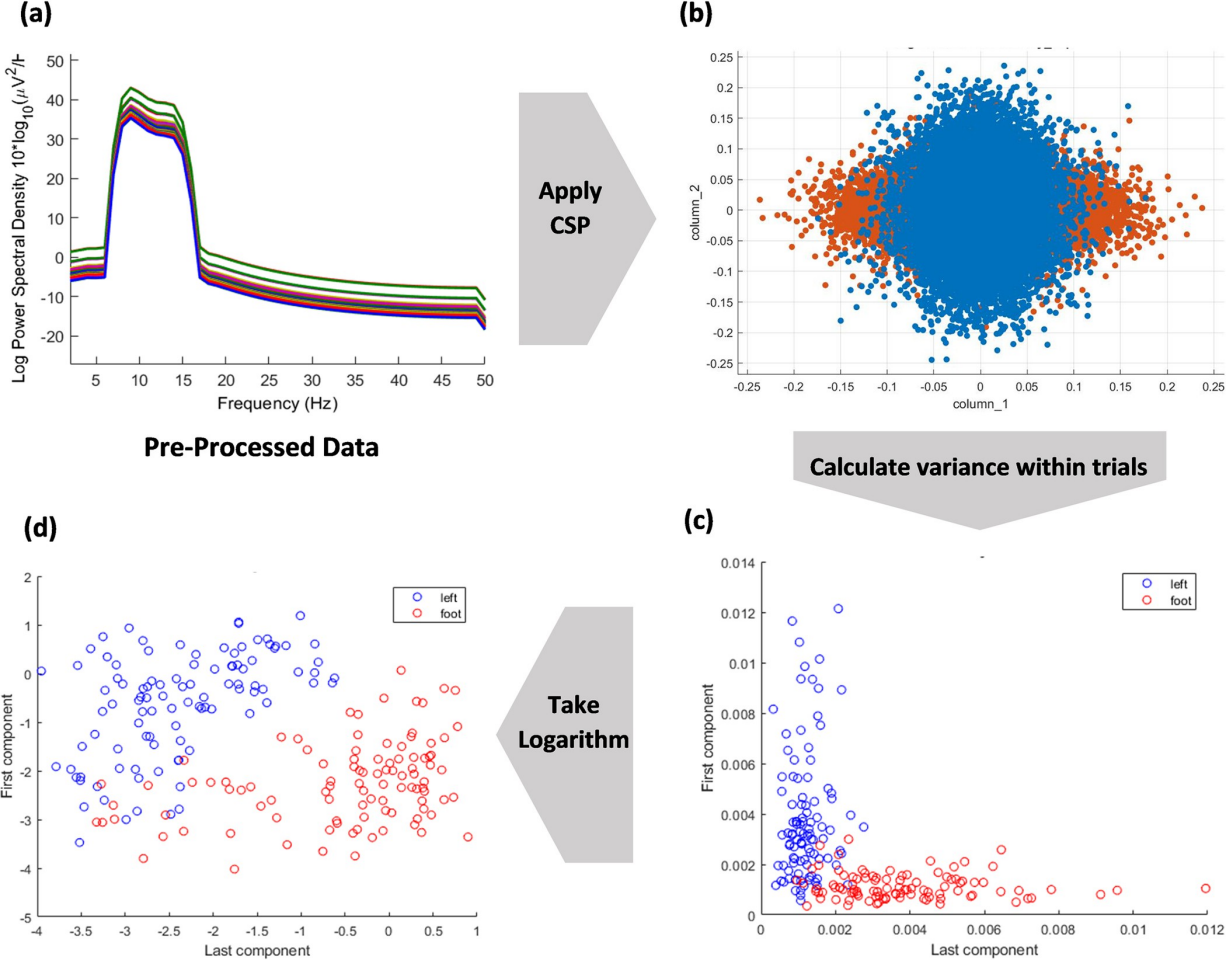
Visual representation of steps involved in feature extraction of preprocessed Data.

Fig 4 shows the visual representation of steps involved in feature extraction of preprocessed data. After applying CSP, a feature vector is obtained by calculating the inter-trial variances of both classes. The logarithm of the obtained results is then computed as given in (11).


$$f=log\;(var(Z_{i}))$$ (11)

Once data has undergone the process of feature extraction, it is ready to enter the stage of classification.

All the analysis during this research was done in MATLAB and some classifiers were implemented through the Classification Learner app of MATLAB.

## Classification

### Support Vector Machine (SVM)

SVM is a robust classifier widely used for both binary [38] and multi-class [39] classification problems. The major motivation for employing SVM for EEG data classification is to address the objective of good generalization by optimizing the performance of the machine while reducing the computational complexity of the learned model, simultaneously [40].

SVM constructs an optimal separating hyperplane for mapping the input vector into a high-dimensional feature space using non-linear mapping [41]. The optimal hyperplane maximizes the distance between itself and the points closest to it, to distinguish data points belonging to various classes.

For binary classification problems, the classifier function f(a) of a training data set {(a_1_, b_1_), (a_2_, b_2_), ……., (a_n_, b_n_)}, is obtained through Lagrange interpolation and is of the form as given by Eq (12):

$$f(a)=sgn(\sum_{i=1}^{N}\omega_{i}b_{i}k(a,a_{i})+c)$$ (12)

Here *k*(*a*, *a*_i_) is a kernel function denoting the dot product between the two entities and *ω*_i_ is the Lagrange operator.

Gaussian Radial Basis Function (RBF) is the kernel function used in this research for applying SVM.

### Logistic regression

Logistic regression is widely used as a classifier for problems where two or more distinct classes or outcomes are to be classified. Logistic regression is very easy to realize and achieves very good performance with linearly separable classes [42]. It finds a wide range of applications in BCI systems where the existence of a feature is predicted based on the set of predictor variables. This algorithm is best suited for models with dichotomous dependent variables [43].

The logistic regression model [44] can be stated as given by (13):

$$Logit\;(P)=ln(\frac{P}{1-P})$$ (13)

Where P is the probability of dichotomous dependent variables related to the predictor variables as given by (14) [44]:

$$ln\left(\frac{P}{1-P}\right)=n_{0}+n_{1}x_{1}+n_{2}x_{2}+\cdots +n_{n}x_{n}$$ (14)

Here, x_1_, x_2,_…, x_n_ are the independent or the predictor variables, whereas n_0_, n_1_, n_2_, ….., n_n_ are the coefficients associated with them.

### Linear Discriminant Analysis (LDA)

LDA is a robust classifier that is simple to implement and has fewer computational requirements, making it ideal for BCI systems. LDA does not modify the data rather provides the best possible decision boundary for distinguishing the given classes, making it one of the best-suited linear classifiers [45].

LDA uses a hyperplane to separate or characterize the data points belonging to two or more classes by maximizing the distance between their means and minimizing the interclass variances, assuming that the data points are linearly separable, and have a normal distribution.

A simple representation of LDA for binary classification problems can be demonstrated through Fisher’s discriminant ratio [46] which finds a projection, ω, to maximize the objective function given in (15):

$$J(\omega )=\frac{|\mu_{1}-\mu_{2}{|}^{2}}{S_{1}{}^{2}+S_{2}{}^{2}}$$ (15)

Here, μ_1_ and μ_2_ are the means of the first and second class respectively and represent the ‘scatter between the class’, whereas S_1_ and S_2_ are the interclass variances and represent the ‘scatter within the class’.

### K- Nearest Neighbour (K-NN)

The k-NN algorithm is a potential classifier for large noisy data as it requires less training and testing time and provides good classification accuracy [12].

The k-NN is a supervised machine learning algorithm that predicts the values of new data points based on how precisely it matches the neighboring points in the training set. It works on the principle of ‘feature similarity’. A value of the nearest data point *k* is chosen. The distance between each point in the test data and each row of training data is estimated using any of the following methods, Euclidean distance, Mahalanobis distance, Minkowski distance, Manhattan distance, etc. [47]. The distances are sorted in ascending orders and the top *k* row is chosen. The most frequent class of these rows is used to assign a class to the test point.

### Naïve Bayes

Naive Bayes is a widely used classifier in BCI systems for detecting binary-class MI tasks as it provides a flexible approach for dealing with any number of classes and is one of the fastest learning algorithms for simultaneously analyzing all its training input [48].

Naïve Bayes is a classification technique that assumes independence among the various features of a class. Naïve Bayes algorithm evaluates a frequency table for the training dataset and then generates a likelihood table by estimating the various probabilities. The posterior probability *P*(*a*|*z*) of the target class ***a*** given the predictor ***z*** is evaluated using Bayes theorem [49] as given in 16:

$$P(y|z)=\frac{P(z|y)P(y)}{P(z)}$$ (16)

Where, P(z| y) represents the likelihood, P(y) represents the prior probability, and P(z) represents the predictor prior probability. The maximum posterior probability among the probabilities of the various classes is the classifier output [50].

### Decision tree

A decision tree is a supervised learning algorithm used for classification problems. One of the major advantages of the decision tree classifier is its ability to use multiple feature subsets and decision rules at different stages of classification [51].

A classification and regression tree (CART) is the most successful method for constructing a Decision Tree [52]. This approach is non-parametric and selects the best attributes for each node of the tree using information gain (IG), Gini diversity index (GDI), and gain ratio [53]. The information gain splits the dataset into segments based on an attribute and assesses how much information a feature provides about a class by evaluating changes in entropy. Based on the value of information gain, the nodes are split and a decision tree is built [54]. The information gain is given by (17).


IG=E(S)–[(WA)*E(F)] (17)

Here, E represents the entropy, S represents the total number of samples, WA represents the weighted average, and F denotes the number of features.

The hyper-parameter settings of the proposed framework are obtained through Bayesian optimization. The optimized hyper-parameters for subject ‘a’ of Dataset 1 are shown below in Table 1.

**Table 1:** Optimized hyper-parameters from subject ‘a’ of dataset 1.

Classifier	Optimization Parameters	
Linear Discriminant	**Discriminant Type**	Linear
	**Iterations**	30
SVM	**Kernel Function**	Gaussian
	**Box Constraint Level**	0.057704
	**Kernel Scale**	0.1–1000
Logistic Regression	-	
Complex Tree	**Maximum Splits**	15
	**Split Criterion**	Maximum deviance reduction
Naïve Bayes	**Distribution Names**	Gaussian
	**Kernel**	Box
KNN	**Number of neighbors**	100
	**Distance Metric**	Mahalanobis
	**Distance Weight**	Inverse

## Results

In this study, a novel framework for the accurate classification of two-class motor imagery EEG signals has been proposed. To evaluate the efficacy of the proposed framework, it has been tested on two publicly available datasets, i.e., BCI Competition IV dataset 1 and BCI Competition III dataset 4a. Different preprocessing methods, feature extraction, and classification techniques have been employed to attain better classification accuracies on both datasets as compared to the accuracies already achieved in literature.

The proposed method employs the filtering of EEG data for each trial using a Butterworth bandpass filter within a frequency range of 8–15 Hz [27]. After attaining the required frequency band, ICA has been applied to extract the useful signals from the EEG data. Furthermore, CSP and log-variance are used for feature extraction. Six different classifiers, i.e., fine Gaussian SVM, fine k-NN, LDA, Gaussian naive Bayes, complex tree, and logistic regression are used to select the best one for classifying the EEG data most accurately. A 10-fold cross-validation was performed and logistic regression outperformed all the other classifiers by attaining an average classification accuracy of 90.42%, followed by LDA with 89.78%, Gaussian naive Bayes with 89.20%, fine Gaussian SVM with 88.14%, fine k-NN with 86.21%, and complex tree with 85.57%, respectively on dataset 1, as demonstrated in Table 2. Logistic regression surpassed other classifiers in terms of achieving the highest accuracies because it is less inclined to overfitting and it is easy to regularize. It doesn’t need tuning and outputs well-calibrated predicted probabilities.

**Table 2:** BCI competition IV dataset 1 classification accuracy (%) with different classifiers.

Subject	Fine KNN	Complex Tree	Logistic Regression	Fine Gaussian SVM	LDA	Naïve Bayes
A	74.50	77.50	82.50	78.00	79.00	79.50
B	76.00	77.00	84.00	76.00	84.50	81.00
C	82.50	82.00	90.50	87.50	89.00	88.50
D	93.00	90.50	92.50	93.50	93.00	92.00
E	92.50	90.00	95.00	94.50	95.50	96.00
F	90.50	91.50	94.50	93.50	92.50	92.00
G	91.50	90.50	94.00	94.00	95.00	95.50
**Mean**	85.78	85.57	90.42	88.14	89.78	89.20

To validate the effectiveness of the proposed method, its performance has been analyzed and compared on dataset 2 as well. Out of all the six classifiers, logistic regression has better performance with the highest classification accuracy among all subjects, with a mean classification accuracy of 95.42%. LDA was second with 95.06%, followed by Gaussian naïve Bayes, fine k-NN, complex tree, and SVM with 94.44%, 92.72%, 90.66%, and 87.78%, respectively. To better comprehend the overall classification using the proposed method on dataset 2, Table 3 shows the individual classification accuracies of each of the 5 subjects and the mean classification accuracy of all the subjects with all the six classifiers.

**Table 3:** BCI competition III dataset 4a classification accuracy (%) with different classifiers.

Subject	Fine KNN	Complex Tree	Logistic Regression	Fine Gaussian SVM	LDA	Naïve Bayes
Aa	89.30	86.40	92.90	84.30	92.50	91.10
Al	98.90	98.20	98.60	96.80	97.10	98.20
Av	80.00	80.40	88.90	77.10	88.60	87.50
Aw	98.60	95.40	99.60	91.40	99.30	98.60
ay	96.80	92.90	97.10	89.30	96.40	96.80
**Mean**	92.72	90.66	95.42	87.78	95.06	94.44

Previously, Li, Y, *et al*. used a cross-correlation technique for feature extraction and logistic regression to classify BCI Competition III dataset 4a and attained an average classification accuracy of 90.29% [55]. In another study, Li, Y, *et al*. improved their method by developing a modified version of the cross-correlation-based logistic regression algorithm and attained an average classification accuracy of 93.91% on the same dataset [56]. The preprocessing of raw data is very important before it is fed into the feature extraction phase. It filters the data from noise and artifacts and results in the improvement of the classification accuracy. In our work, ICA has been employed to clean the data from artefactual components, which has a significant contribution in obtaining higher average classification accuracy i.e., 95.42% on the same dataset compared to methods proposed earlier.

**Fig 5:**
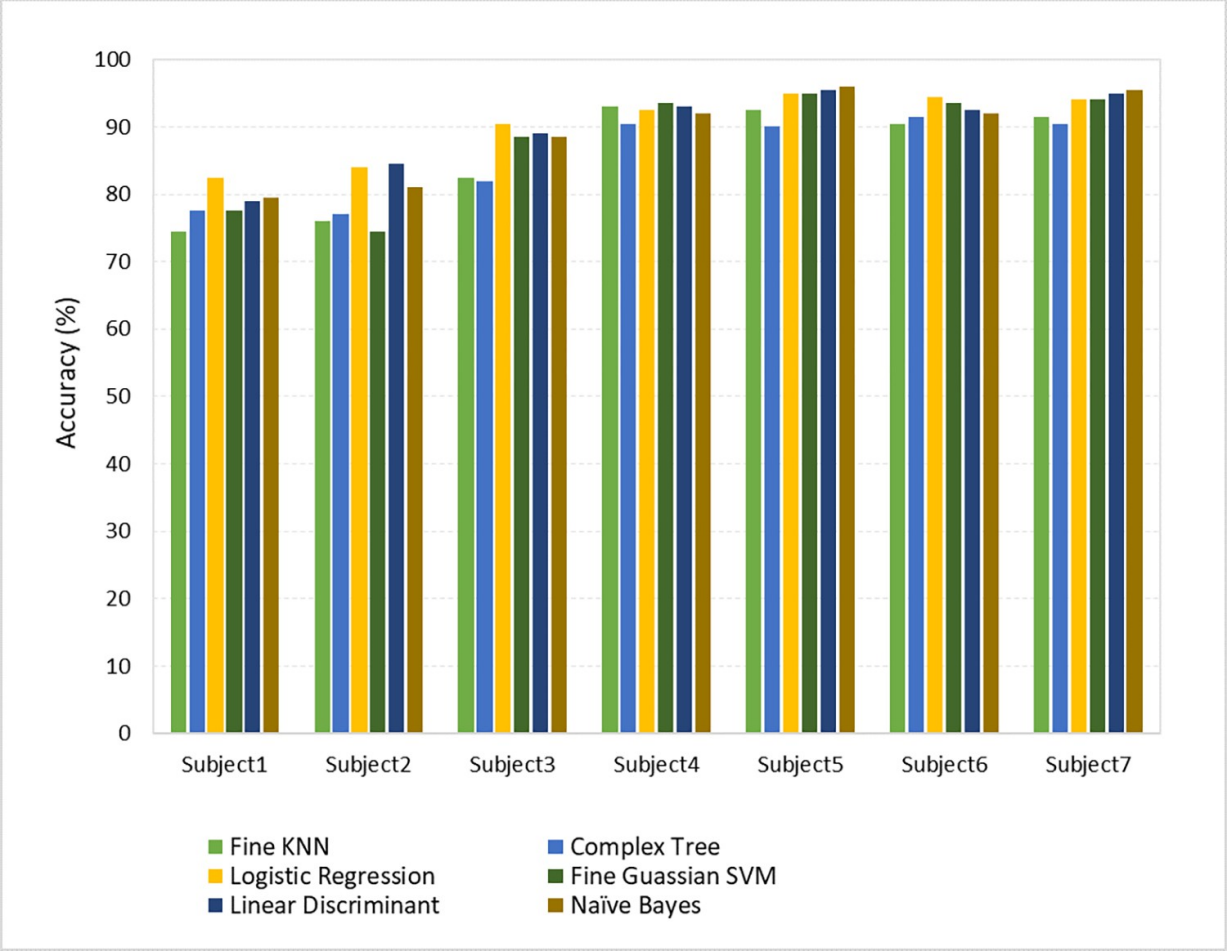
Comparison of classification accuracies of dataset 1 with different classifiers.

**Fig 6:**
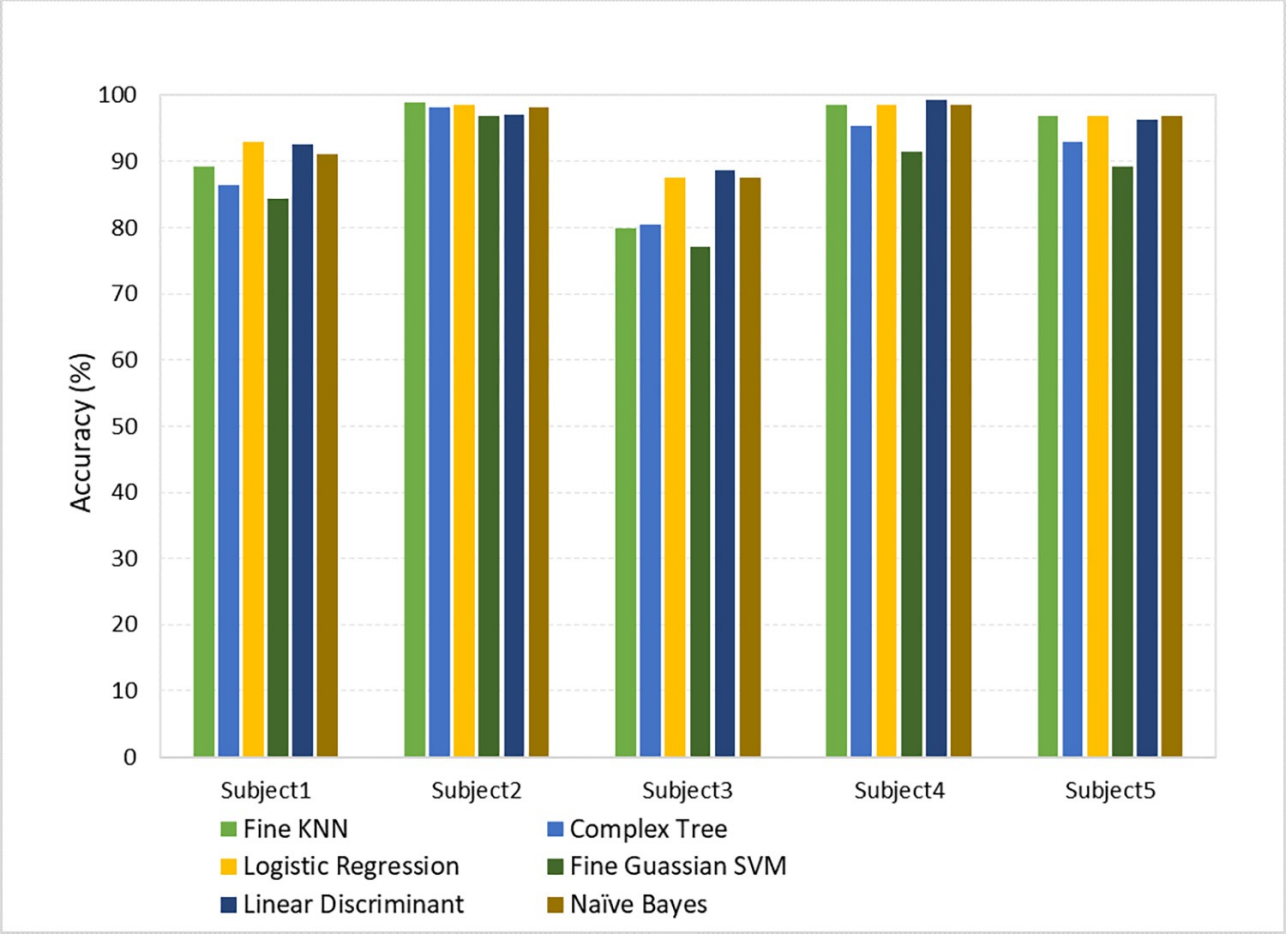
Comparison of classification accuracies of dataset 2 with different classifiers.

To evaluate the performance of various classifiers on dataset 1 and dataset 2, two histograms have been computed as shown in Figs 5 and 6, respectively.

The classification results of the two datasets suggest that using a bandpass filter and ICA for preprocessing, along with CSP, log-variance, and logistic regression, gives better classification accuracies. Average accuracy that has been attained on dataset 1 is 90.42%, while on dataset 2 is 95.42%, when logistic regression has been used for classification. Commendable accuracy for both datasets has been achieved as ICA removes the ocular artifacts from the data, which is the most considerable noise in EEG. A combination of CSP and log-variance has proved to be very effective. Moreover, logistic regression has less computational complexity, and is very efficient to train for linearly separable data.

Tables 4 and 5 lists the classification accuracies of all the methods previously employed on dataset 1 and dataset 2, respectively, along with the classification accuracies attained by our proposed method.

**Table 4:** BCI competition IV dataset 1 classification accuracy (%) of dataset 1 with proposed method compared with other methodologies.

	Jin, Miao, & al., 2019	Miao, Yin, & al., 2019	Fu & al., 2020	Zhang & al., 2020	Zhang & al., 2020	Proposed Method
Subjects	CCS-RCSP	R-AdaBoost	RCSP-RDA	CSP-WPD+LOG	CSP-FB+LOG	ICA-CSP+logvar with Logistic Regression
A	85.50	83.50	97.00	64.00	73.00	82.50
B	67.00	62.00	96.00	69.00	75.00	84.50
C	_	_	72.50	80.00	87.00	90.50
D	_	_	75.00	95.00	98.00	92.50
E	_	_	78.50	100.00	99.00	95.00
F	79.50	76.50	96.00	93.00	93.00	94.50
G	94.50	91.50	95.50	94.00	97.00	94.00
**Mean**	81.60	78.40	87.21	85.00	88.86	90.42

**Table 5:** BCI competition III dataset 4a classification accuracy (%) of dataset 2 with proposed method compared with other methodologies.

	Su et al., 2020	Miao et al., 2021	Chen et al., 2020	Kirar et al., 2020	Wijaya et al., 2020	Proposed Method
Subject	DCNMF	CTFSP with SVM	CSP with SVM	GQCSP with SVM	LRFS+TSD	ICA-CSP+log-var with Logistic Regression
Aa	76.43	86.07	86.52	82.59	93.93	92.90
Al	98.21	98.57	98.89	97.19	92.14	98.60
Av	72.35	52.14	67.86	75.94	98.57	88.90
Aw	75.43	96.07	90.14	98.20	94.64	99.60
Ay	66.46	92.14	90.89	99.00	96.79	97.10
**Mean**	77.78	85.00	86.86	90.58	95.21	95.42

In comparison to the approaches proposed in the recent research methodologies, our method with ICA as the preprocessing method, CSP & log-variance as the feature extraction techniques, and logistic regression as the classifier, is able to effectively improve the classification accuracy of motor imagery signals to a remarkable extent. In a nutshell, our proposed method is a promising scheme for the improvement of the classification performance of MI-based BCI.

## Discussion

In this research, binary-class MI EEG signals have been classified using a novel framework with a logistic regression classification algorithm. The method employs a bandpass filter and ICA for preprocessing, CSP and log-variance for feature extraction, and six different classifiers to select the best one for effective classification of EEG signals. The bandpass filter has reduced the noise interference, ICA has revealed the patterns of active brain regions enabling effective source estimation for accurate identification of MI and played a significant role in extracting useful information, and CSP & log-variance has helped in discriminating intent patterns and extracting spatial information from the signal. After selecting time-window segments, frequency filtering, and feature extraction, the selected features are fed into the classifier. Out of various classifiers used, logistic regression has proved to be the simplest yet very powerful for classifying two-class motor imagery EEG signals very efficiently.

The proposed method is evaluated by dataset 1 of the BCI competition IV and dataset 4a of the BCI Competition III. Both the datasets are validated by using 10-fold cross-validation. Table 2 shows the classification accuracies of all 7 subjects of dataset 1. The results show that maximum classification accuracy of 90.42% is achieved with a logistic regression classifier. Table 3 lists the results of the classification of individual subjects for dataset 2. For dataset 2 as well, logistic regression has surpassed all the other classifiers in terms of generating the highest average classification accuracy of around 95.42%. As compared to the existing methods for both datasets, the proposed method showed excellent classification performance. Tables 4 and 5 demonstrate that our proposed method has effectively improved the average classification accuracy rate of BCI Competition IV dataset 1 by 1.56% and by 0.21% for BCI Competition III dataset 4a, respectively, as compared to the accuracies attained by the previous researchers.

The key advantages of the proposed framework include the improved pre-processing, optimized feature extraction, and the reduced classification complexity due to the use of Logistic Regression as a classifier, which reduces the execution time. The main advantages of the proposed framework are discussed below.

As the EEG signals are a mixture of both cerebral and artefactual sources. Most of the previous studies only employ the application of a bandpass filter and do not involve the removal of other artifacts from the EEG data. But in this study, we have focused on removing EOG artifacts from the data. For this purpose, ICA has been applied. ICA has successfully removed ocular artifacts (artifacts related to eye movement) from the meaningful data and contributed to EEG signal enhancement. ICA decomposes the observed signals into independent components, and once the components are extracted from the original signal, the clean signal is reconstructed by disregarding ICs contained artifacts. The application of ICA on the EEG data after the applying the bandpass filter gives us an edge over the methods proposed in previous studies and is one of the key factors contributing towards a better average classification accuracy.

Another factor contributing to higher classification accuracy is the use of CSP for feature extraction and logistic regression for classification. The CSP feature extraction technique employs a frequency selection approach to identify the most significant features associated with the motor imagery task. Logistic regression classifier is one of the basic classification algorithms for binary classification problems. It is relatively simple to execute, interpret, and train. Unlike decision trees or support vector machines, it allows models to be easily updated to reflect new data. In low-dimensional space, logistic regression is less inclined to overfitting. Moreover, it doesn’t require input features to be scaled, is easy to regularize, doesn’t need tuning, and outputs well-calibrated predicted probabilities, all of which are the major factors for outperforming other classifiers.

The first proposed method without proper pre-processing of the signals failed to provide any extraordinary results. So, in order to obtain significant results, all the filtering and pre-processing techniques that are a part of the second proposed framework must be implemented on the raw data to remove artifacts and obtain a specific band of frequencies before moving towards the feature extraction and classification stages.

## Conclusion

This paper introduces a novel approach for improving the classification accuracy of binary-class EEG data. The importance of ICA for preprocessing of raw EEG data and the power of CSP for extracting the appropriate signal characteristics from extraneous data has been highlighted. Moreover, the classification performance of various classifiers has been analyzed and compared in this paper. The analysis has shown that the classification performance of logistic regression with ICA as a preprocessor and CSP & log-variance as the feature extraction techniques is more efficient than SVM, LDA, decision trees, naïve Bayes, and k-NN. With logistic regression, an average classification accuracy of 90.42% is achieved on BCI Competition IV dataset 1, and 95.42% on BCI Competition III dataset 4a, which are comparable to all the research methodologies proposed earlier on both datasets.

One of the limitations of CSP is that it can only apply to binary classification problems. For multi-class problems, some modifications to the basic CSP algorithm are required. Moreover, CSP filters are prone to noise and overfitting, and if a subject has a lesser number of training samples, the performance of CSP is much reduced as compared to the subjects with a larger number of training samples. A concern for the use of ICA for preprocessing is that it suffers from order ambiguity and normalization. Furthermore, logistic regression cannot be used for non-linear problems and is prone to overfitting for classification problems in which the number of features is greater than the number of observations.

In the future, we aim to optimize the proposed method even more by applying new and improved preprocessing techniques and by selecting a more appropriate time window, to make it more feasible for real time implementation. Application of improved classification methodologies, development of algorithms for optimized channel selection, and multi-class classification expansion will be our future approaches.

## Supporting information

S1 File This is the code file which can be utilized to generate results shown in the research paper. (RAR)
